# INTERCEPT‐AD: Understanding the Patient Experience and Expectations for Treatment Through Qualitative Interviews Among Trial Participants with Early Alzheimer’s Disease and Their Study Partners

**DOI:** 10.1002/alz.089519

**Published:** 2025-01-09

**Authors:** Kelly Johnston, Carrie Presnall, Elizabeth Merikle, Stephanie Cline, Todd Feaster, Eric Siemers

**Affiliations:** ^1^ Fortrea, Inc., Durham, NC USA; ^2^ Acumen Pharmaceuticals, Charlottesville, VA USA

## Abstract

**Background:**

Incorporation of the patient voice into drug development has been recognized as fundamental; recent legislation has addressed the importance of collecting patient experience data. Qualitative patient interviews conducted in tandem with clinical trials, often in later phases, are among the most common means of eliciting these data. To assess aspects of patient experience earlier in development, we conducted semi‐structured qualitative interviews following participation in the phase 1 ACU‐001 (INTERCEPT‐AD) trial, a study evaluating the safety and tolerability of the Aβ oligomer‐targeting monoclonal antibody ACU193, among a subset of participants with mild cognitive impairment (MCI) or mild Alzheimer’s disease (AD) and their study partners. Interview topics included disease experience and expectations for treatment.

**Method:**

Interviews followed the end‐of‐study visit and included both participants and study partners. Participants were asked to report their most problematic symptoms, to describe what they would hope to gain from a new AD treatment, and what changes would be meaningful. The interview guide was developed in accordance with FDA Patient Focused Drug Development guidance. Principles of qualitative thematic analysis guided the coding and analysis of transcripts, with additional features drawn from grounded theory, conforming to best practices.

**Result:**

Twenty‐eight participants and their study partners were interviewed. Participants reported a broad array of problems consistent with AD. The most frequent included difficulty with memory or cognitive functioning (96.4%), getting lost (71.4%), mood (60.7%), and difficulty communicating (57.1%). Within each of these groupings, specific problems varied among participants; higher numbers of participants reported forgetting appointments, names, and losing things (81.4%) and getting lost while driving (90.0%), while fewer reported feeling isolated (5.9%) and experiencing confusion (11.1%). Nearly every participant desired treatment that would keep the disease from getting worse or slow progression (89.3%), which was equated with maintenance of functional abilities. Maintaining the ability to recognize loved ones and communicate were also stated as important benefits of a new treatment.

**Conclusion:**

Problems reported by participants were typical to AD, although there was heterogeneity in overall AD experience. Changes in mood were frequently reported. These findings highlight the need to contextualize clinical trial results by incorporating the patient voice.

